# Endotrophin Levels Are Associated with Allograft Outcomes in Kidney Transplant Recipients

**DOI:** 10.3390/biom13050792

**Published:** 2023-05-05

**Authors:** Nadja Sparding, Federica Genovese, Daniel Guldager Kring Rasmussen, Morten A. Karsdal, Nicoline V. Krogstrup, Marie Bodilsen Nielsen, Mads Hornum, Subagini Nagarajah, Henrik Birn, The CONTEXT Study Group, Bente Jespersen, Martin Tepel, Rikke Nørregaard

**Affiliations:** 1 Nordic Bioscience, 2730 Herlev, Denmark; 2 Biomedical Sciences, Faculty of Health and Medical Science, University of Copenhagen, 2200 Copenhagen, Denmark; 3 Department of Renal Medicine, Aarhus University Hospital, 8200 Aarhus, Denmark; 4 Department of Biomedicine, Aarhus University, 8000 Aarhus, Denmark; 5 Department of Nephrology, Rigshospitalet and Department of Clinical Medicine, University of Copenhagen, 2200 Copenhagen, Denmark; 6 Department of Nephrology, Odense University Hospital, 5000 Odense, Denmark; 7 Institute of Molecular Medicine, Cardiovascular and Renal Research, University of Southern Denmark, 5000 Odense, Denmark; 8 Department of Clinical Medicine, Aarhus University, 8000 Aarhus, Denmark

**Keywords:** biomarkers, fibrosis, delayed graft function, kidney transplantation, kidney failure

## Abstract

Early prediction of kidney graft function may assist clinical management, and for this, reliable non-invasive biomarkers are needed. We evaluated endotrophin (ETP), a novel non-invasive biomarker of collagen type VI formation, as a prognostic marker in kidney transplant recipients. ETP levels were measured with the PRO-C6 ELISA in the plasma (P-ETP) of 218 and urine (U-ETP/Cr) of 172 kidney transplant recipients, one (D1) and five (D5) days, as well as three (M3) and twelve (M12) months, after transplantation. P-ETP and U-ETP/Cr at D1 (P-ETP AUC = 0.86, *p* < 0.0001; U-ETP/Cr AUC = 0.70, *p* = 0.0002) were independent markers of delayed graft function (DGF) and P-ETP at D1 had an odds ratio of 6.3 (*p* < 0.0001) for DGF when adjusted for plasma creatinine. The results for P-ETP at D1 were confirmed in a validation cohort of 146 transplant recipients (AUC = 0.92, *p* < 0.0001). U-ETP/Cr at M3 was negatively associated with kidney graft function at M12 (*p* = 0.007). This study suggests that ETP at D1 can identify patients at risk of delayed graft function and that U-ETP/Cr at M3 can predict the future status of the allograft. Thus, measuring collagen type VI formation could aid in predicting graft function in kidney transplant recipients.

## 1. Introduction

Although kidney transplantation improves patient prognosis compared to dialysis, allograft function eventually declines, leading to the need for dialysis or the requirement for a new transplant. Better predictors of a successful transplant and graft fibrosis are required to monitor kidney transplant recipients for individual treatment [1,2]. One step towards improved outcomes and better decisions tailored to the individual patient may be the use of novel biomarkers.

Here we propose endotrophin (ETP), a novel non-invasive biomarker of collagen type VI (COL VI) formation, as a tool to monitor and predict the status of the allograft over time. COL VI is one of the major components of the renal stroma of the fibrotic kidney [3]. Renal fibrosis is seen in a large proportion of kidney allografts within six months and increasingly at two years following transplantation [4], and the imbalance in tissue turnover is an essential part of chronic kidney transplant rejection [5]. Assessment of the extent and formation of COL VI in the kidney would be a valuable tool, as tubulointerstitial fibrosis is the best predictor of progression to kidney failure [6], and COL VI is predominantly localized in the tubulointerstitial matrix. The PRO-C6 (C-terminal C5 domain of type VI collagen α3 chain released by BMP-1) assay measures the C-terminal of COL VI, which is cleaved off from newly assembled tetramers after their release in the extracellular space and may therefore be used as a measure of COL VI formation. Interestingly, the released fragment also contains ETP, a bioactive fragment derived from the proteolytic cleavage of the C-terminal fragment. ETP has been associated with pro-fibrotic and pro-inflammatory properties in animal and cell models of fibrosis [7,8,9]. High levels of ETP in plasma may therefore identify patients with a high level of COL VI formation that, due to the effects of ETP, are more likely to have increased inflammation (e.g., macrophage infiltration) and fibrogenesis (e.g., enhanced TGFβ signaling), e.g., in the transplanted kidney. Previously, it has been shown that pretransplant ETP can predict delayed graft function [10] and that ETP is associated with graft failure and mortality [11].

To further evaluate the potential of ETP as a biomarker of poor outcomes in kidney transplantation we measured ETP in the plasma of 218 and urine of 172 kidney transplant recipients enrolled in the trial cohort “Remote Ischemic Conditioning in Renal Transplantation—Effect on Immediate and Extended Kidney Graft Function (CONTEXT)” [12] at different time points after transplantation. Our hypothesis is that ETP is associated with the onset of kidney graft function and with kidney graft function at twelve months. A prospective, observational cohort including 146 kidney transplant recipients was used as a validation cohort for the association of ETP with delayed graft function.

## 2. Materials and Methods

### 2.1. Clinical Cohorts

The CONTEXT cohort (NCT01395719), enrolled patients receiving a kidney transplant from a deceased donor were used to test whether remote ischemic conditioning (RIC) could improve outcomes after renal transplantation [12,13]. The CONTEXT cohort was approved by the national data protection agencies and ethical committees in the countries involved (Denmark: The National Committee on Health Research Ethics; Sweden: Regional Ethical Board; the Netherlands: METc UMCG). Blood and urine samples were collected just before transplantation (baseline, BL) and one (D1) and five (D5) days and three (M3) and twelve (M12) months after transplantation. EDTA-blood and urine samples were collected, centrifuged at 2800× *g* at 4 °C for 10 min within 2 h, aliquoted in 1 mL vials, and stored at −80 °C. The immunosuppressive regimen included induction with intravenous basiliximab and methylprednisolone or corresponding oral doses of prednisolone followed by oral tacrolimus, mycophenolate mofetil, and prednisolone [14].

The prospective, observational “Monitoring after kidney transplantation (MoMoTx)” cohort was used as a validation cohort. MoMoTx includes 146 incident patients receiving a kidney allograft in a single center. The study was approved by the ethical committee of the Region of Southern Denmark (Project-ID: 8-20100098). Blood and urine samples were collected at D1. Heparin plasma was prepared by centrifugation (1620× *g* for 4 min) of blood samples within 2 h of obtaining the samples, aliquoted in 1 mL vials, and stored at −70 °C. Induction therapy and immunosuppressive therapy (including basiliximab, tacrolimus, and mycophenolate mofetil) were administered according to the local protocol. Recipients with ABO-incompatible donors (17% of the MoMoTx patients) received rituximab, immunoabsorption, tacrolimus, mycophenolate mofetil, and prednisolone before transplantation [15]. The baseline characteristics of kidney transplant recipients and donors were retrieved from medical records.

In both cohorts, delayed graft function (DGF) was defined by at least one session of dialysis within seven days of transplantation [15,16].

Informed and written consent was obtained prior to inclusion and the two studies were performed in adherence to the Declarations of Helsinki and Istanbul.

### 2.2. Biochemical Analysis

We measured ETP in the EDTA plasma (P-ETP) of 218 patients and in urine (U-ETP/Cr) of 172 patients enrolled in the CONTEXT cohort at BL, D1, D5, M3, and M12. Data from BL were previously described [10] and only included in this work to study the pre- to post-transplantation change of ETP. Data from D5 were only used to observe the changes in the biomarkers over time and were not used for any further analysis, due to a lack of other measurements (i.e., plasma creatinine (P-Cr) and urinary albumin-to-creatinine ratio (U-ACR)) at the same time point. In addition, we measured ETP in heparin plasma and urine of 146 kidney transplant recipients at D1 in the prospective, observational cohort MoMoTx. There is a strong correlation between ETP levels measured in EDTA and heparin plasma when measured in matched samples from healthy subjects (Figure S1, Supplementary Materials).

ETP was measured with the PRO-C6 competitive enzyme-linked immunosorbent assay (ELISA) developed at Nordic Bioscience (Denmark). The PRO-C6 assay detects the 10 amino acids at the C-terminal of the α3 chain of COL VI. The technical characteristics and procedure of the assay have been previously described [17]. The concentration of ETP in urine was normalized using the concentration of urinary creatinine (mg/mL) measured using the QuantiChromTM Creatinine Assay Kit (BioAssay Systems, Hayward, CA, USA) to account for urine output.

In CONTEXT, P-Cr (µmol/L) was measured twice daily for the first week and twice weekly until 30 days after transplantation. If dialysis was needed post-transplantation, P-Cr was measured twice a week until 30 days after dialysis. The time to a 50% reduction in P-Cr (tCr50) was calculated by modeling the changes in P-Cr for each patient as previously described [12,18]. U-ACR (mg/g) was measured at the local Department of Clinical Biochemistry using automated, standard clinical assays. Estimated GFR (eGFR) was calculated using the simplified Modification of Diet in Renal Disease (MDRD) creatinine-based equation [19] without correction for race, as >90% of the included patients were Caucasian.

### 2.3. Statistical Analysis

In the CONTEXT cohort, P-ETP and U-ETP/Cr were not normally distributed at any time point. The biomarker values were log_2_-transformed, and all parametric statistical analyses were performed on log_2_-transformed data. Non-parametric statistical analyses were performed on untransformed data.

Clearance of creatinine and ETP was calculated: Cr clearance = (U-Cr/P-Cr) × (U-volume/collection time) and ETP clearance = (U-ETP/P-ETP) × (U-volume/collection time).

Spearman correlation was used to study the correlation of P-ETP and U-ETP/Cr with P-Cr, U-ACR, and eGFR, as well as the correlation between P-ETP and U-ETP/Cr at the different time points.

To analyze the prognostic value of P-ETP and U-ETP/Cr to predict future kidney function, multiple linear regression using log_2_(P-ETP) or log_2_(U-ETP/Cr) and log_2_(P-Cr) was used to determine the association of the markers at different time points with eGFR at M3 and M12, as well as the change of eGFR from M3 to M12 (delta eGFR = (eGFR at M3 − eGFR at M12)/eGFR at M3).

To analyze the prognostic value of P-ETP and U-ETP/Cr to predict DGF, we observed the area under the receiver operating characteristics (AUROC) curve for D1 P-ETP as well as D1 U-ETP/Cr in both the discovery and validation cohorts. C-statistics were used to compare receiver operating characteristics (ROC) curves. The odds ratio (OR) of log_2_(P-ETP) and log_2_(U-ETP/Cr) were calculated using logistic regression. For the analysis of tCr50, we excluded patients with primary non-function. Cox-proportional hazard regression analysis was used to analyze the association of P-ETP and U-ETP/Cr at D1 with tCr50.

## 3. Results

### 3.1. Baseline Characteristics of the Cohorts

The recipient and transplant characteristics of the analyzed patients in CONTEXT and MoMoTx are reported in Table 1.

### 3.2. Change in Endotrophin over Time

**Figure 1:**
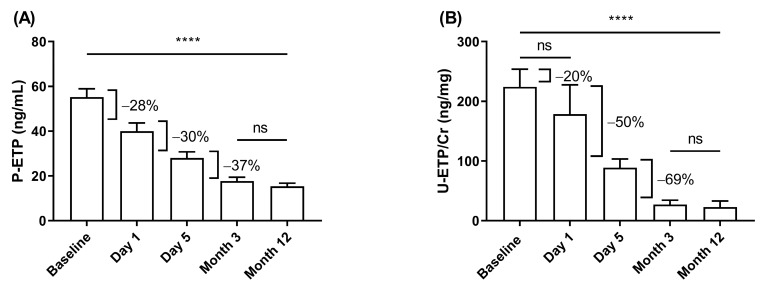
Change in ETP levels in CONTEXT. Change in (**A**) P-ETP and (**B**) U-ETP/Cr over time before (baseline) and after transplantation. Data are presented as mean ± 95% CI. Statistical differences were assessed with paired ANOVA test (A: *n* = 61 and B: *n* = 34) and Kruskal–Wallis (all samples); *p*-values (**** *p* < 0.0001) indicate the statistical difference between all groups except those marked as ns (not significant).

In CONTEXT, P-ETP and U-ETP/Cr levels decreased by 20–28% from BL to D1, 30–50% from D1 to D5 after transplantation, and stabilized between D5 and M3 after transplantation (Figure 1). The decrease was more marked in urine than in plasma.

To evaluate the effect of restored filtration on ETP levels, the clearance of P-ETP at D1 was compared to the clearance of P-Cr at D1. For both P-ETP (r = −0.44, *p* < 0.0001) and P-Cr (r = −0.60, *p* < 0.0001) measured at D1, clearance decreased with increasing biomarker levels (Figure S2, Supplementary Materials).

### 3.3. Effect of Remote Ischemic Conditioning

In the CONTEXT study, a randomized controlled intervention study with an RIC and a sham-RIC group, there was no effect of RIC on patient outcomes as previously published [12,13]. Hence, it seems reasonable to combine the data and to exclude any effect of the RIC on ETP levels, and we compared ETP levels in the RIC and sham-RIC groups. In our study, we found no difference in P-ETP and U-ETP/Cr levels at any time point in patients stratified by RIC (Table 2). Therefore, all subsequent analyses were performed on pooled data from the RIC and the sham-RIC group in the CONTEXT study.

### 3.4. Association of Endotrophin with Kidney Graft Function at Different Time Points

In CONTEXT, P-ETP correlated positively with P-Cr at every time point. P-ETP correlated with U-ACR at D1 but not at M12 (U-ACR at M3 was not available) and it correlated inversely with eGFR at M3 and M12. U-ETP/Cr correlated positively with P-Cr and U-ACR and negatively with eGFR at all time points where such measurements were available. In addition, P-ETP correlated with U-ETP/Cr at every time point (Table 3).

### 3.5. Prognostic Value of Endotrophin for Future Kidney Graft Function

When measured at M3 in CONTEXT, U-ETP/Cr was significantly and negatively associated with kidney function (eGFR) at M12 independently from P-Cr. In addition, U-ETP/Cr at M3 was associated with the change in eGFR from M3 to M12 (delta eGFR). P-ETP measured at M3 was not associated with kidney function (eGFR) at M12 or with delta eGFR (Table 4).

### 3.6. Prognostic Value of Endotrophin for Delayed Graft Function

**Figure 2:**
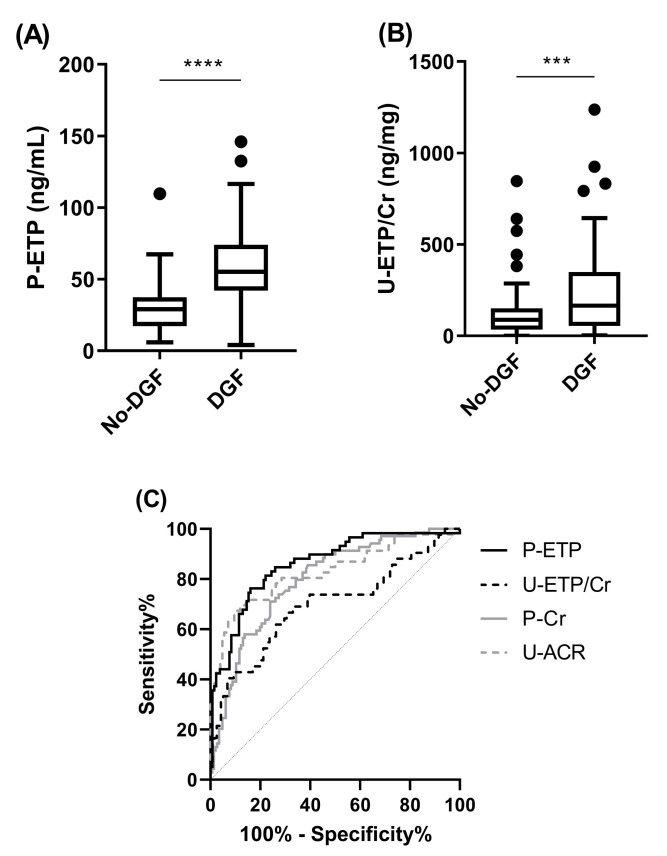
Delayed graft function in CONTEXT. Levels of (**A**) P-ETP and (**B**) U-ETP/Cr at D1 in patients from CONTEXT without DGF and with DGF. (**C**) ROC curve comparison for DGF for P-ETP, U-ETP/Cr, P-Cr, and U-ACR at D1. Statistical differences between groups were assessed by Mann-Whitney; *** *p* < 0.001, **** *p* < 0.0001.

In CONTEXT, 70 patients (32.1%) required dialysis during the first week after transplantation (DGF). P-ETP at D1 was more elevated in patients experiencing DGF compared to no-DGF (Figure 2A) and was a good discriminator of patients with DGF (area under the curve (AUC) = 0.86; *p* < 0.0001; Figure 2C and Table 5). U-ETP/Cr (AUC = 0.70, *p* = 0.0002), P-Cr (AUC = 0.80, *p* < 0.0001), and U-ACR (AUC = 0.83, *p* < 0.0001) were also able to discriminate patients with DGF when measured at D1 (Figure 2B,C and Table 5). P-ETP at D1 was a better discriminator than P-Cr at D1 (comparison of ROC curves, *p* = 0.006; Table 5). When patients were stratified according to D1 ETP levels into quartiles, higher P-ETP (AUC = 0.83, *p* < 0.0001) and U-ETP/Cr (AUC = 0.68, *p* = 0.0003) levels were prognostic for DGF (Table 5).

In a multiple logistic regression analysis including P-Cr, both P-ETP and U-ETP/Cr at D1 were independently associated with risk of DGF at D1 with an OR of 6.3 (*p* < 0.0001) and 1.5 (*p* = 0.006), respectively (Table 5).

### 3.7. Association with 50% Reduction in Plasma Creatinine

The time to 50% reduction in plasma creatinine, tCr50, is a measure of the time for kidney graft recovery [12,18]. In CONTEXT, P-ETP at D1 was significantly negatively associated with tCr50, independently of P-Cr at D1 (Table 6).

### 3.8. Validation of the Prognostic Value of Endotrophin for Delayed Graft Function

Samples from MoMoTx, a prospective, observational cohort, were included to validate the D1 ETP data obtained in the CONTEXT cohort. The transplant recipients in the MoMoTx validation cohort had significantly lower age and D1 P-Cr as well as higher body mass index. In contrast to CONTEXT, MoMoTx included living donors and the donor age was significantly higher in the CONTEXT cohort (Table 1). The levels of P-ETP and U-ETP/Cr at D1 were significantly lower in the transplant recipients in the MoMoTx cohort compared to the recipients in the CONTEXT cohort (Table 1).

**Figure 3:**
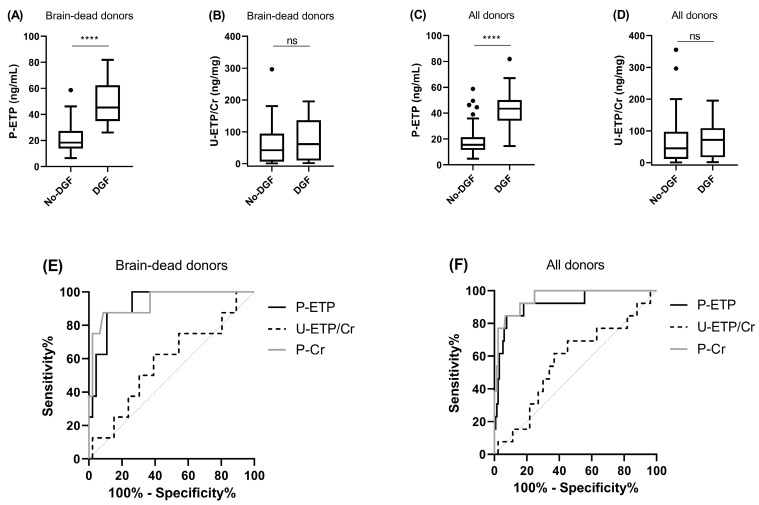
Delayed graft function in MoMoTx. Levels of (**A**,**C**) P-ETP and (**B**,**D**) U-ETP/Cr measured at D1 in kidney transplant recipients from brain-dead and all donors in MoMoTx. ROC curve comparison for DGF for P-ETP and U-ETP/Cr at D1 in kidney transplant recipients from (**E**) brain-dead donors and (**F**) all donors. All donors include living and brain-dead donors. Statistical differences between groups were assessed by Mann-Whitney; **** *p* < 0.0001, ns = not significant.

In the MoMoTx cohort, recipients receiving transplants from both brain-dead (*n* = 54) and living donors (*n* = 92) were included. A total of 13 of the 146 transplant recipients (8.9%) experienced DGF and 8 of these received transplants from brain-dead donors. Levels of P-ETP at D1 were significantly higher in transplant recipients experiencing DGF (Figure 3A,C), and P-ETP at D1 had a good discriminatory power (deceased donors AUC = 0.92, *p* < 0.0001, Figure 3E and all donors AUC = 0.93, *p* < 0.0001, Figure 3F), confirming the findings in the CONTEXT cohort (Figure 2A,C). In MoMoTx, the ROC curves for DGF for D1 P-ETP were not significantly different from the ROC curves for D1 P-Cr (deceased donors *p* = 0.88, and all donors *p* = 0.35). U-ETP/Cr at D1 was unable to predict DGF in the validation cohort (deceased donors AUC = 0.58, *p* = 0.49, Figure 3E, and all donors AUC = 0.57, *p* = 0.42, Figure 3F).

## 4. Discussion

Our main findings in this study were: (i) ETP measured in plasma one day after transplantation was prognostic for DGF in both the CONTEXT and MoMoTx cohort, independently from plasma creatinine. In addition, plasma ETP was associated with the speed of P-Cr decline as evaluated by tCr50, suggesting that patients with lower day one P-ETP levels recovered kidney graft function more rapidly, and (ii) ETP measured in urine at three months after transplantation was independently associated with kidney function at twelve months and with the change in kidney function between three months and twelve months after transplantation.

From our data, we can conclude that measuring the same markers at different time points during the course of transplantation and in different matrices may provide different information. Early after transplantation (one and five days after transplantation), ETP may reflect restoration of filtration; three months after transplantation, ETP levels are significantly lower, but still prognostic for future graft function in the CONTEXT study (twelve months after transplantation).

Non-invasive biomarkers that can inform on the risk of post-transplantation problems are needed. Here we evaluated ETP, a biomarker of COL VI formation previously associated with poor outcomes in chronic kidney disease (CKD) patients [3,20,21], in the plasma and urine of kidney transplant recipients, to evaluate its prognostic potential as a biomarker of allograft outcome.

The main results described in this study are based on the CONTEXT cohort. Here we showed that patients with higher levels of both P-ETP and U-ETP/Cr one day after transplantation were at higher risk of experiencing DGF. Moreover, patients with low levels of P-ETP at D1 (independently of plasma creatinine levels at D1) were able to recover functionality in the transplanted kidney (time to halving of P-Cr) significantly faster than patients with high levels of P-ETP.

The exact mechanism for this association is unclear. Since the levels of ETP in both plasma and urine can be affected by the recovered GFR in the graft, as suggested by the strong correlation with D1 P-Cr, we could consider D1 ETP a marker of filtration. ETP levels at D1 were approximately twice as high in CONTEXT compared to MoMoTx, which was likely mostly related to the fact that CONTEXT only included deceased donors, whereas MoMoTx also included living donors with known faster onset of function. It remains to be investigated whether the contribution to the plasma pool of ETP fragments from the donor kidney can be significant and related to the health of the transplanted organ, as these data may indicate.

When looking at the multivariate logistic regression analyses for the risk of DGF, the prognostic ability of P-ETP at D1 as well as U-ETP/Cr at D1 were independent of P-Cr, despite the strong correlation of the ETP with P-Cr. This suggests that the clinical utility of ETP goes beyond the information provided by established markers of kidney function (GFR). This aspect, as well as the suggested contribution of the donor organ to the marker levels, needs further corroboration and understanding in future studies.

The MoMoTx cohort validated the prognostic value of ETP measured one day after transplantation for DGF. Investigating the prognostic value of ETP in cohorts with different recipient and donor characteristics gives an indication of whether the biomarker can be used in a heterogeneous population. The MoMoTx and CONTEXT cohorts were quite different in terms of transplant recipient characteristics (age, BMI, P-Cr, and ETP levels) and donor characteristics (type and age). Urinary ETP at D1 was not able to predict DGF in MoMoTx, which may be explained by the lower levels of U-ETP/Cr at D1 in MoMoTx compared to CONTEXT combined with the lower sensitivity of urinary compared to plasma levels of ETP. Nevertheless, plasma ETP measured at D1 was still able to predict DGF, which highlights the robustness of the biomarker.

ETP measured in both plasma and urine three months after transplantation may reflect the fibrosis activity in the transplanted kidney, at a time at which the hemodynamics of the new organ are stable. Here we showed that high levels of ETP in urine were associated with lower kidney function at twelve months and with a decline in kidney function between three and twelve months after transplantation. Kidney fibrosis has been proposed as one of the main promoters of the progression of CKD to kidney failure [6], and tubulointerstitial fibrosis and tubular atrophy are common findings in biopsies of renal allografts after transplantation [4]. We have previously established a link between the levels of ETP in both the serum and urine of patients with CKD and fibrosis in the renal tissue [22], and we have shown how COL VI deposition is markedly increased in the kidneys of patients with evident fibrosis [3]. Moreover, we have shown, in studies using populations with different CKD etiologies, that high levels of ETP in serum and urine are prognostic for a detrimental outcome [3,20,21,23]. ETP is a signaling molecule derived from the processing of the C-terminal end of the COL VI α3 chain (detected by PRO-C6) and has been associated with pro-inflammatory and pro-fibrotic cell recruitment and activation [8,24]. It is therefore tempting to associate the deleterious effects of ETP with the increased risk of kidney function deterioration, likely triggered by an increase in kidney fibrosis, seen in patients with high levels of excreted ETP.

Several biomarkers are currently being evaluated to monitor the status of the renal allograft after transplantation. Plasma neutrophil gelatinase-associated lipocalin (NGAL), a biomarker vastly explored in the context of acute kidney injury, was proposed as a prognostic marker for loss of graft function when measured 3 months after transplantation [25]. In the CONTEXT cohort, it was previously shown [26] that plasma NGAL measured at D1 showed a good prognostic potential for DGF, but it failed to show an association with long-term graft function. In this study, ETP showed both good prognostic potential for DGF and an association with long-term graft function. Nielsen et al. also evaluated the prognostic values of liver-type fatty acid-binding protein (U-L-FABP), U-cystatin C, and U-chitinase-3-like protein 1 (U-YKL-40) for DGF but showed only a weak correlation and may not be useful to predict DGF [26]. Fibroblast growth factor 23 (FGF23) has been evaluated in kidney transplant recipients in several studies reporting conflicting results. While four reports showed plasma FGF23 to be prognostic for mortality [27], another study failed to find an independent association between FGF23 and death and loss of allograft function [28]. As with FGF23, the prognostic potential of other biomarkers, such as soluble CD30 [29,30] and collectin liver 1 (CL-L1) [31], in kidney transplant recipients remains unclear. Taken together this indicates that ETP has significant prognostic potential to monitor the status of the allograft and identify the patients at higher risk of having DGF when measured one day after transplantation, as well as to predict long-term outcomes when measured three months after transplantation. This could be of importance for future intervention studies for the prolongation of kidney graft function.

This study has some limitations: there were no available graft biopsies to confirm that the soluble levels of ETP at M3 after transplantation are associated with fibrosis development in the graft. Moreover, it is at present challenging to confirm that ETP could reflect ongoing fibrosis and that it is not just a marker of filtration, given its high association with P-Cr at all studied time points. The data will need to be replicated in independent cohorts with available biopsies.

Further exploration of the incremental predictive value of ETP to the routinely assessed variables currently used to monitor kidney transplant recipients is needed in order to consider the possibility of adding this test to the currently used models of risk prediction in transplantation. If this marker proves to be of significant utility in the evaluation of the kidney transplant recipient, an advantage of ETP is that it is tested through a simple ELISA platform, which is easy to perform and can be readily implemented and maintained in centralized clinical laboratories.

In conclusion, in this study we have for the first time described the prognostic potential of ETP as a biomarker to identify the patients at higher risk of DGF when measured one day after transplantation and as a biomarker to monitor the future status of the allograft when measured three months after transplantation.

## Figures and Tables

**Table 1:** Baseline characteristics of kidney transplant recipients in the CONTEXT cohort and in the MoMoTx cohort.

Characteristics	Parameter	CONTEXT		MoMoTx		Comparison of Cohorts
		*n*	Median (IQR)	*n*	Median (IQR)	*p*-Value
Recipient	Age (years)	218	59 (49–66)	146	49 (40–59)	<0.0001
	Sex (female), *n* (%)	218	84 (39)	146	50 (34)	
	BMI (kg/m^2^)	193	25.1 (23.0–27.6)	146	26.3 (23.8–29.1)	0.012
	BL U-ACR (mg/g)	152	687 (276–1875)	NA	NA	
	BL P-Cr (µmol/L)	216	638 (498–756)	NA	NA	
	BL P-ETP (ng/mL)	211	50.7 (36.6–65.5)	NA	NA	
	BL U-ETP (ng/mg)	111	207.1 (115.5–278.8)	NA	NA	
	D1 P-Cr (µmol/L)	216	523 (348–675)	146	363 (257–545)	<0.0001
	D1 P-ETP (ng/mL)	193	34.8 (21.3–52.5)	146	17.3 (11.7–26.0)	<0.0001
	D1 U-ETP/Cr (ng/mg)	163	104.4 (40.6–196.6)	146	48.6 (11.7–97.4)	<0.0001
Transplant	Donor age (years)	218	58 (52–65)	146	53 (45–60)	<0.0001
	Donor female, *n* (%)	218	99 (45)	146	80 (56)	
	Number of HLA mismatches	218	3 (3–4)	146	3 (2–4)	
	Kidney from, *n* (%):	218		146		
	- Brain-dead donor		197 (90)		54 (37)	
	- Circulatory-dead donor		21 (10)		-	
	- ABO-I living donor		-		25 (17)	
	- ABO-C living donor		-		67 (46)	
	Center, *n* (%):	218		146		
	- Aarhus		130 (60)		-	
	- Gothenburg		44 (20)		-	
	- Groningen		23 (10)		-	
	- Rotterdam		21 (10)		-	
	- Odense		-		146 (100)	

Values are presented as median with interquartile range (IQR) for continuous variables and *n* (%) for categorical variables. Differences between the two groups were analyzed with the two-tailed Mann–Whitney U test. ABO-C, ABO-compatible; ABO-I, ABO-incompatible; BL, baseline (before transplantation); BMI, body mass index; D1, one day after transplantation; HLA, human leukocyte antigen; P-Cr, plasma creatinine; P-ETP, plasma endotrophin; U-ACR urinary albumin to creatinine ratio; U-ETP/Cr, urine endotrophin/creatinine.

**Table 2:** Levels of P-ETP and U-ETP/Cr in patients receiving RIC or Sham-RIC at different time points in CONTEXT.

		D1		D5		M3		M12	
	Treatment	*n*	Median (95% CI)	*n*	Median (95% CI)	*n*	Median (95% CI)	*n*	Median (95% CI)
P-ETP (ng/mL)	RIC	94	35.2 (30.4–39.7)	98	23.8 (19.5–27.3)	86	13.6 (12.4–14.9)	79	13.0 (11.9–14.5)
	Sham-RIC	97	33.6 (29.1–40.0)	97	20.9 (17.4–25.3)	89	14.8 (13.4–16.6)	76	13.7 (12.8–15.3)
	*p*-value		0.77		0.35		0.09		0.31
U-ETP/Cr (ng/mg)	RIC	80	102.8 (72.9–129.4)	83	66.1 (46.7–83.8)	83	8.8 (5.4–13.8)	82	4.2 (3.2–5.8)
	Sham-RIC	80	100.3 (78.8–136.0)	87	66.5 (47.1–84.7)	84	8.4 (5.6–13.3)	79	5.1 (3.9–7.7)
	*p*-value		0.83		0.96		0.87		0.30

Two patients were excluded from the analysis since RIC or sham-RIC was not reported. Kruskall–Wallis test for difference between P-ETP and U-ETP/Cr levels with or without ischemic pre-conditioning. CI, confidence interval; D1, one day after transplantation; D5, five days after transplantation; M3, three months after transplantation; M12, twelve days after transplantation; P-ETP, plasma endotrophin; RIC, remote ischemic conditioning; U-ETP/Cr, urine endotrophin/creatinine.

**Table 3:** Correlation table for P-ETP and U-ETP/Cr with P-Cr, U-ACR, and eGFR at the different time points in CONTEXT.

		ETP					
		D1		M3		M12	
		rho	*p*-Value	rho	*p*-Value	rho	*p*-Value
P-ETP	P-Cr	0.669	<0.0001	0.549	<0.0001	0.477	<0.0001
	U-ACR	0.468	<0.0001	NA	NA	0.145	0.10
	eGFR	NA	NA	−0.584	<0.0001	−0.520	<0.0001
U-ETP/Cr	P-Cr	0.254	0.001	0.285	0.0002	0.332	<0.0001
	U-ACR	0.236	0.003	NA	NA	0.332	0.0001
	eGFR	NA	NA	−0.318	<0.0001	−0.385	<0.0001
	P-ETP	0.377	<0.0001	0.355	<0.0001	0.338	<0.0001

Spearman rank correlation. eGFR, estimated glomerular filtration rate; D1, one day after transplantation; M3, three months after transplantation; M12, twelve days after transplantation; P-Cr, plasma creatinine; P-ETP, plasma endotrophin; U-ACR, urinary albumin to creatinine ratio; U-ETP/Cr, urine endotrophin/creatinine.

**Table 4:** Association of ETP with future eGFR in CONTEXT.

Multivariate Linear Regression		ETP								
		D1			M3			M12		
Variables	Outcome	*n*	r_partial_	*p*-Value	*n*	r_partial_	*p*-Value	*n*	r_partial_	*p*-Value
P-ETP, P-Cr	eGFR M3	179	0.04	0.56	171	−0.17	0.028			
	eGFR M12	172	0.05	0.54	165	−0.15	0.054	154	−0.08	0.36
	Delta eGFR(M3 to M12)				165	−0.01	0.86			
U-ETP/Cr, P-Cr	eGFR M3	152	−0.01	0.88	166	−0.07	0.37			
	eGFR M12	147	−0.05	0.58	160	−0.21	0.007	159	0.03	0.67
	Delta eGFR(M3 to M12)				160	0.21	0.008			

The reported r_partial_ and *p*-values are for P-ETP or U-ETP/Cr. D1, one day after transplantation; eGFR, estimated glomerular filtration rate; M3, three months after transplantation; M12, twelve months after transplantation; P-Cr, plasma creatinine; P-ETP, plasma endotrophin; U-ETP/Cr, urine endotrophin/creatinine.

**Table 5:** Prognostic analyses for DGF in CONTEXT. AUC for P-ETP, U-ETP/Cr, P-Cr, U-ACR, and quartiles of ETP at D1 for DGF. Odds ratio for log_2_(P-ETP) and log_2_(U-ETP/Cr) in univariate and multivariate logistic regression (including log_2_(P-Cr)) for DGF.

	AUROC Analysis				Logistic Regression			
					Univariate		Multivariate	
	*n* (% DGF)	AUC (95% CI)	*p*-Value	Comparison of ROC Curves	OR (95% CI)	*p*-Value	OR (95% CI)	*p*-Value
D1 P-ETP	190 (31.1)	0.86 (0.80–0.91)	<0.0001	D1 P-Cr: *p* = 0.006	7.7 (4.0–14.7)	<0.0001	6.3 (3.0–13.1)	<0.0001
D1 U-ETP/Cr	160 (26.3)	0.70 (0.62–0.77)	0.0002	D1 P-Cr: *p* = 0.15	1.4 (1.1–1.8)	0.002	1.5 (1.1–1.9)	0.006
D1 P-Cr	215 (32.1)	0.80 (0.74–0.85)	<0.0001					
D1 U-ACR	172 (26.7)	0.83 (0.76–0.88)	<0.0001	D1 P-Cr: *p* = 0.67				
Quartiles of D1 P-ETP, median (IQR)	190 (31.1)	0.83 (0.77–0.88)	<0.0001					
Q1: 15.0 (13.2–17.6)	47 (2.1)							
Q2: 29.2 (26.1–31.3)	48 (14.6)							
Q3: 40.5 (37.1–45.3)	47 (36.2)							
Q4: 64.6 (57.6–81.7)	48 (70.8)							
Quartiles of D1 U-ETP/Cr, median (IQR)	160 (26.3)	0.68 (0.60–0.75)	0.0003					
Q1: 14.6 (5.2–29.9)	41 (17.5)							
Q2: 70.2 (53.9–87.7)	41 (10.0)							
Q3: 139.9 (124.9–156.4)	40 (30.0)							
Q4: 278.5 (229.5–572.6)	41 (47.5)							

Three patients were excluded from the analysis since DGF or no-DGF was not reported. AUC, area under the curve; AUROC, area under the receiver operating characteristics; CI, confidence interval; D1, one day after transplantation; DGF, delayed graft function; IQC, interquartile range; OR, odds ratio; P-Cr, plasma creatinine; P-ETP, plasma endotrophin; ROC, receiver operating characteristics; U-ACR urinary albumin to creatinine ratio; U-ETP/Cr, urine endotrophin/creatinine.

**Table 6:** Cox proportional hazard regression analysis for time to a 50% decrease in plasma creatinine in CONTEXT. The univariate analysis included log_2_(P-ETP) or log_2_(U-ETP/Cr). The multivariate analysis included log_2_(P-ETP) or log_2_(U-ETP/Cr) and log_2_(P-Cr).

	Univariate		Multivariate	
	HR (95% CI)	*p*-Value	HR (95% CI)	*p*-Value
D1 P-ETP	0.66 (0.55–0.79)	<0.0001	0.75 (0.60–0.95)	0.01
D1 U-ETP/Cr	0.94 (0.87–1.00)	0.06	0.96 (0.89–1.03)	0.27

CI, confidence interval; D1, one day after transplantation; HR, hazard ratio; P-ETP, plasma endotrophin; U-ETP/Cr, urine endotrophin/creatinine.

## Data Availability

The data that support the findings of this study are available from the corresponding author upon reasonable request.

## Supplementary Materials

The following supporting information can be downloaded at: https://www.mdpi.com/article/10.3390/biom13050792/s1, Figure S1: Correlation between heparin and EDTA plasma ETP levels; Figure S2: Biomarker clearance in CONTEXT.

## References

[1] Schold J.D., Kaplan B. (2010). The Elephant in the Room: Failings of Current Clinical Endpoints in Kidney Transplantation. Am. J. Transplant..

[2] Schold J.D., Srinivas T.R., Howard R.J., Jamieson I.R., Meier-Kriesche H.-U. (2008). The Association of Candidate Mortality Rates With Kidney Transplant Outcomes and Center Performance Evaluations. Transplantation.

[3] Rasmussen D.G.K., Fenton A., Jesky M., Ferro C., Boor P., Tepel M., Karsdal M.A., Genovese F., Cockwell P. (2017). Urinary Endotrophin Predicts Disease Progression in Patients with Chronic Kidney Disease. Sci. Rep..

[4] Boor P., Floege J. (2015). Renal Allograft Fibrosis: Biology and Therapeutic Targets. Am. J. Transplant..

[5] Lai X., Zheng X., Mathew J.M., Gallon L., Leventhal J.R., Zhang Z.J. (2021). Tackling Chronic Kidney Transplant Rejection: Challenges and Promises. Front. Immunol..

[6] Barbour S.J., Reich H.N. (2012). Risk Stratification of Patients with IgA Nephropathy. Am. J. Kidney Dis..

[7] Sun K., Park J., Gupta O.T., Holland W.L., Auerbach P., Zhang N., Marangoni R.G., Nicoloro S.M., Czech M.P., Varga J. (2014). Endotrophin Triggers Adipose Tissue Fibrosis and Metabolic Dysfunction. Nat. Commun..

[8] Lee C., Kim M., Lee J.H., Oh J., Shin H.-H., Lee S.M., Scherer P.E., Kwon H.M., Choi J.H., Park J. (2019). COL6A3-Derived Endotrophin Links Reciprocal Interactions among Hepatic Cells in the Pathology of Chronic Liver Disease. J. Pathol..

[9] Sun K., Park J., Kim M., Scherer P.E. (2017). Endotrophin, a Multifaceted Player in Metabolic Dysregulation and Cancer Progression, Is a Predictive Biomarker for the Response to PPARγ Agonist Treatment. Diabetologia.

[10] Tepel M., Alkaff F.F., Kremer D., Bakker S.J.L., Thaunat O., Nagarajah S., Saleh Q., Berger S.P., van den Born J., Krogstrup N.V. (2022). Pretransplant Endotrophin Predicts Delayed Graft Function after Kidney Transplantation. Sci. Rep..

[11] Kremer D., Alkaff F.F., Post A., Knobbe T.J., Tepel M., Thaunat O., Berger S.P., van den Born J., Genovese F., Karsdal M.A. (2022). Plasma Endotrophin, Reflecting Tissue Fibrosis, Is Associated with Graft Failure and Mortality in KTR: Results from Two Prospective Cohort Studies. Nephrol. Dial. Transplant..

[12] Krogstrup N.V., Oltean M., Nieuwenhuijs-Moeke G.J., Dor F.J.M.F., Møldrup U., Krag S.P., Bibby B.M., Birn H., Jespersen B. (2017). Remote Ischemic Conditioning on Recipients of Deceased Renal Transplants Does Not Improve Early Graft Function: A Multicenter Randomized, Controlled Clinical Trial. Am. J. Transplant..

[13] Nielsen M.B., Krogstrup N.V., Oltean M., Nieuwenhuijs-Moeke G.J., Dor F.J.M.F., Birn H., Jespersen B. (2019). Remote Ischaemic Conditioning and Early Changes in Plasma Creatinine as Markers of One Year Kidney Graft Function-A Follow-up of the CONTEXT Study. PLoS ONE.

[14] Krogstrup N.V., Oltean M., Bibby B.M., Nieuwenhuijs-Moeke G.J., Dor F.J.M.F., Birn H., Jespersen B. (2015). Remote Ischaemic Conditioning on Recipients of Deceased Renal Transplants, Effect on Immediate and Extended Kidney Graft Function: A Multicentre, Randomised Controlled Trial Protocol (CONTEXT). BMJ Open.

[15] Borst C., Xia S., Bistrup C., Tepel M. (2015). Interleukin-8 Transcripts in Mononuclear Cells Determine Impaired Graft Function after Kidney Transplantation. PLoS ONE.

[16] Yarlagadda S.G., Coca S.G., Garg A.X., Doshi M., Poggio E., Marcus R.J., Parikh C.R. (2008). Marked Variation in the Definition and Diagnosis of Delayed Graft Function: A Systematic Review. Nephrol. Dial. Transplant..

[17] Sun S., Henriksen K., Karsdal M.A., Byrjalsen I., Rittweger J., Armbrecht G., Belavy D.L., Felsenberg D., Nedergaard A.F. (2015). Collagen Type III and VI Turnover in Response to Long-Term Immobilization. PLoS ONE.

[18] Krogstrup N.V., Bibby B.M., Aulbjerg C., Jespersen B., Birn H. (2016). A New Method of Modelling Early Plasma Creatinine Changes Predicts 1-Year Graft Function after Kidney Transplantation. Scand. J. Clin. Lab. Investig..

[19] Levey A.S., Coresh J., Greene T., Stevens L.A., Zhang Y., Hendriksen S., Kusek J.W., Van Lente F. (2006). Using Standardized Serum Creatinine Values in the Modification of Diet in Renal Disease Study Equation for Estimating Glomerular Filtration Rate. Ann. Intern. Med..

[20] Fenton A., Jesky M.D., Ferro C.J., Sørensen J., Karsdal M.A., Cockwell P., Genovese F. (2017). Serum Endotrophin, a Type VI Collagen Cleavage Product, Is Associated with Increased Mortality in Chronic Kidney Disease. PLoS ONE.

[21] Rasmussen D.G.K., Hansen T.W., von Scholten B.J., Nielsen S.H., Reinhard H., Parving H.-H., Tepel M., Karsdal M.A., Jacobsen P.K., Genovese F. (2018). Higher Collagen VI Formation Is Associated With All-Cause Mortality in Patients With Type 2 Diabetes and Microalbuminuria. Diabetes Care.

[22] Sparding N., Genovese F., Rasmussen D.G.K., Karsdal M.A., Neprasova M., Maixnerova D., Satrapova V., Frausova D., Hornum M., Bartonova L. (2021). Endotrophin, a Collagen Type VI-Derived Matrikine, Reflects the Degree of Renal Fibrosis in Patients with IgA Nephropathy and in Patients with ANCA-Associated Vasculitis. Nephrol. Dial. Transplant..

[23] Frimodt-Møller M., Hansen T.W., Rasmussen D.G.K., Theilade S., Nielsen S.H., Karsdal M.A., Genovese F., Rossing P. (2019). A Marker of Type VI Collagen Formation (PRO-C6) Is Associated with Higher Arterial Stiffness in Type 1 Diabetes. Acta Diabetol..

[24] Bu D., Crewe C., Kusminski C.M., Gordillo R., Ghaben A.L., Kim M., Park J., Deng H., Xiong W., Liu X.-Z. (2019). Human Endotrophin as a Driver of Malignant Tumor Growth. JCI Insight.

[25] Jafari A., Khatami M.R., Dashti-Khavidaki S., Lessan-Pezeshki M., Abdollahi A. (2017). Plasma Neutrophil Gelatinase-Associated Lipocalin as a Marker for Prediction of 3-Month Graft Survival after Kidney Transplantation. Int. J. organ Transplant. Med..

[26] Nielsen M.B., Krogstrup N.V., Nieuwenhuijs-Moekeid G.J., Oltean M., Dor F.J.M.F., Jespersen B., Birn H. (2019). P-NGAL Day 1 Predicts Early but Not One Year Graft Function Following Deceased Donor Kidney Transplantation—The CONTEXT Study. PLoS ONE.

[27] Pichler G., Haller M.C., Kainz A., Wolf M., Redon J., Oberbauer R. (2016). Prognostic Value of Bone- and Vascular-Derived Molecular Biomarkers in Hemodialysis and Renal Transplant Patients: A Systematic Review and Meta-Analysis. Nephrol. Dial. Transplant..

[28] Bienaimé F., Dechartres A., Anglicheau D., Sabbah L., Montgermont P., Friedlander G., Ravaud P., Legendre C., Prié D. (2017). The Association Between Fibroblast Growth Factor 23 and Renal Transplantation Outcome Is Modified by Follow-up Duration and Glomerular Filtration Rate Assessment Method. Kidney Int. Rep..

[29] Chen Y., Tai Q., Hong S., Kong Y., Shang Y., Liang W., Guo Z., He X. (2012). Pretransplantation Soluble CD30 Level As a Predictor of Acute Rejection in Kidney Transplantation. Transplant. J..

[30] Rajakariar R., Jivanji N., Varagunam M., Rafiq M., Gupta A., Sheaff M., Sinnott P., Yaqoob M. (2005). High Pre-Transplant Soluble CD30 Levels Are Predictive of the Grade of Rejection. Am. J. Transplant..

[31] Smedbråten J., Sagedal S., Åsberg A., Hartmann A., Rollag H., Mjøen G., Fagerland M.W., Hansen S.W.K., Mollnes T.E., Thiel S. (2017). Collectin Liver 1 and Collectin Kidney 1 of the Lectin Complement Pathway Are Associated With Mortality After Kidney Transplantation. Am. J. Transplant..

