# Supplementary material for: Endotrophin Levels Are Associated with Allograft Outcomes in Kidney Transplant Recipients
Source: Biomolecules. 2023 May 5;13(5):792. doi: 10.3390/biom13050792 (PMC10216383; doi:10.3390/biom13050792)
Supplement: Supplementary file 1 [file biomolecules-13-00792-s001.zip › biomolecules-2372139-supplementary.pdf]

Supplementary Materials

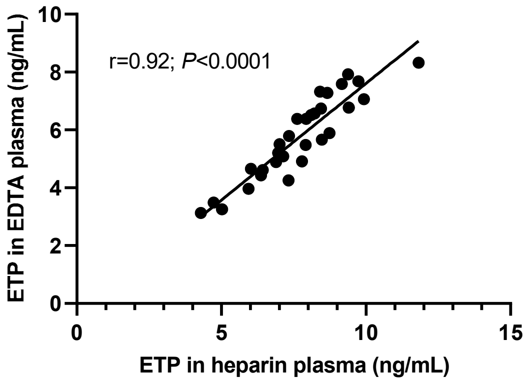

Figure S1. Correlation between heparin and EDTA plasma ETP levels. Spearman correlation between ETP measured in heparin and EDTA plasma. The correlation is based on matched samples from 30 healthy subjects.

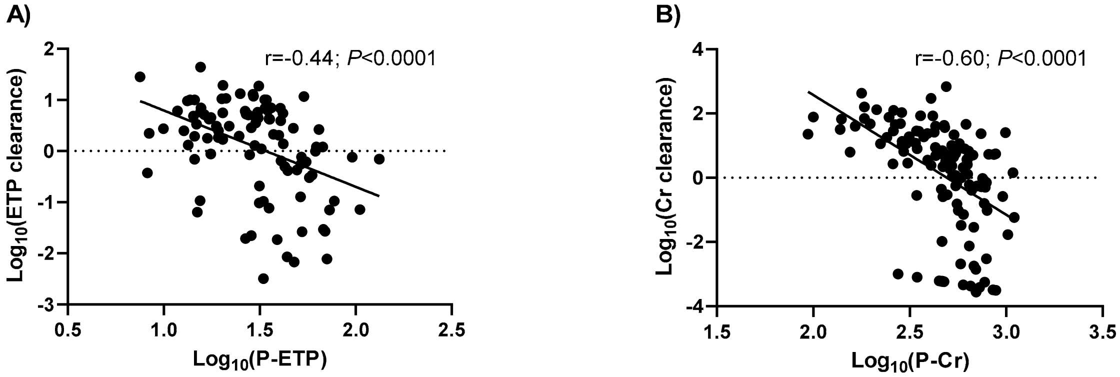

Figure S2. Biomarker clearance in CONTEXT. Clearance of (A) endotrophin and (B) creatinine at D1 in patients from CONTEXT.
